# Could Tack-Curing Influence Margin Continuity and Conversion Degree of a Universal Dual-Curing Cement?

**DOI:** 10.3390/ma18122920

**Published:** 2025-06-19

**Authors:** Leila Es Sebar, Andrea Baldi, Allegra Comba, Isabella Sannino, Leonardo Iannucci, Sabrina Grassini, Tolou Shokuhfar, Nicola Scotti

**Affiliations:** 1 Department of Applied Science and Technology, Politecnico di Torino, 10129 Turin, Italy; isabella.sannino@polito.it (I.S.); leonardo.iannucci@polito.it (L.I.); sabrina.grassini@polito.it (S.G.); 2 Department of Surgical Science, Università degli Studi di Torino, 10124 Turin, Italy; andrea.baldi@unito.it (A.B.); allegra.comba@unito.it (A.C.); 3 Department of Biomedical Engineering, University of Illinois, Chicago, IL 60607, USA; tolou@uic.edu

**Keywords:** micro-CT, Raman spectroscopy, marginal adaptation, degree of conversion, dual luting cement

## Abstract

Proper polymerization protocol is crucial for the long-term success of full-ceramic crown restorations. This study investigates the margin continuity and degree of conversion (DC) of a universal dual-curing cement under full-ceramic crowns subjected to different polymerization protocols and thermal aging. Intact human upper central incisors and canines were prepared for crowns, digitally designed, and milled from reinforced lithium silicate (Celtra Duo, Dentsply). Crowns were cemented using a universal dual-curing cement (G-Cem One, GC) with two polymerization protocols: (G1) microbrush excess removal, 1 min waiting, and 20 s light curing per side; (G2) 5 s tack curing per side, excess removal with a scaler, and 20 s light curing. Marginal adaptation was assessed using micro-computed tomography, and DC was evaluated with Raman spectroscopy before and after artificial thermal aging (10,000 cycles between 5 °C and 55 °C). Statistical comparisons were performed with significance set at *p* < 0.05. Results showed significantly poorer marginal adaptation in the tack-curing group, with no post-aging differences between groups. Baseline DC was high in all samples, with no protocol-dependent variations; nevertheless, aging increased DC in G1. These findings highlight the importance of selecting an appropriate polymerization protocol to ensure optimal marginal adaptation and polymerization efficiency.

## 1. Introduction

Glass–ceramic (GC) materials are renowned for their ability to mimic the appearance and optical properties of natural teeth. These ceramics are particularly suitable for creating various all-ceramic dental restorations. When combined with adhesive cements, they enable dental professionals to employ a minimally invasive technique, which leads to more conservative restorations while providing outstanding aesthetic and functional results. In this field, resin-based cements are crucial for the adhesive bonding of a range of indirect dental restorations [1,2,3,4,5]. However, a major challenge in this context is the limited light transmission through ceramics, which can compromise the polymerization of the underlying cement. This limitation has driven the development of dual-curing resin-based cements, which are specifically designed to blend the benefits of both chemical and light-cure polymerization. Their formulation includes photo-initiators that facilitate controlled polymerization and chemical initiators, allowing them to complete the curing process even when direct light exposure is obstructed, thus ensuring a satisfactory degree of conversion (DC) [6,7]. The effectiveness of these systems, however, depends not only on their formulation but also on the clinical curing technique employed [6,8,9,10].

Simultaneously, curing techniques must be designed to enable clinicians to effectively handle the luting cementation process and eliminate any excess cement from the edges of the restoration [1,4,11,12,13]. To address this, some researchers have suggested an initial brief light cure, lasting between 3 and 8 s, commonly referred to as a “tack-cure” [1,14,15,16]. This approach aims to transform the cement into a semi-gel state, making it easier to remove excess material with a curette or scalpel. Stegall et al. suggest that tack-curing assists the cement in reaching an optimal state for polymerization [17]. However, based on the early vitrification theory, immediately initiating light curing could create a cross-linked polymer network that traps free radicals, thereby hindering the chemical reaction and reducing the final degree of conversion [18].

Additionally, there are concerns regarding the integrity and fit of margins following various light-curing techniques, particularly when sharp tools are used to remove excess material. Indeed, the cementation process should prevent poor marginal adaptation, which has been shown to heighten the risk of restorative and periodontal complications [4,19,20,21,22,23].

Recently, there has been an introduction of “universal” dual-curing systems, which are touted as versatile for use with or without a primer, contingent upon clinical requirements [24]. Several studies have already examined their adhesive characteristics and bond strength [25]. Nonetheless, there remains a paucity of literature regarding their effectiveness, specifically concerning marginal adaptation and the degree of conversion when used for luting GC crowns with varying light-curing techniques [26]. Therefore, the present study aimed to evaluate the effects of different light-curing protocols on the marginal adaptation and degree of conversion of a universal dual-curing cement used to lute GC crowns. Integrating micro-CT and Raman spectroscopy (RS), these parameters were analyzed before and after thermal aging. The null hypotheses tested were that the marginal adaptation (1) and the degree of conversion (2) of universal dual-curing cement in GC crowns remain unaffected by different light-curing methods and the thermal aging process.

## 2. Materials and Methods

### 2.1. Sample Selection and Preparation

A set of 48 human upper central incisors and canines, extracted within the past 3 months for periodontal or orthodontic reasons, were selected for micro-CT and RS analysis. Selected specimens had to match the following characteristics: no carious lesions, demineralization, abrasion, or cracks visible under 6× optical magnification and transillumination, intact cement–enamel junction (CEJ). Selected samples, after debridement with an ultrasonic device, were stored in a Chloramine 0.5% solution at 4 °C. All samples were collected with informed consent in the Department of Cariology and Operative Dentistry, University of Turin. The ethical committee of the University of Turin approved the study protocol (DS_00071_2018).

An experienced operator (more than 15 years of practice in prosthodontics) submitted all the specimens to an anatomical full-crown chamfer preparation (1 mm thickness in axial walls and 1.5 mm on the occlusal surface) using a dedicated diamond bur mounted on a parallelometer (878K-016-314 FG Maillefer, Dentsply Sirona, NC, USA), positioning the finish line 1 ± 0.5 mm above CEJ. After that, samples were individually digitalized using an intraoral scanner (Cerec Omnicam, Dentsply Sirona, NC, USA), and restorations with identical anatomy and thickness were designed with computer-assisted design (CAD) software (Cerec System 5.1, Dentsply Sirona, NC, USA). According to the selected material, an A3 (VITA scale) reinforced lithium silicate (Celtra Duo, Dentsply Sirona, NC, USA), the thickness of all crowns was set to 1.5 mm. This was meant to both respect manufacturer instructions and optimize light transmission through the material [7]. The cement offset was set equal to 80 μm in the axial walls and occlusal area, while no spacing was given in the chamfer line. All restorations were produced with the same chairside milling device (CEREC MC XL, Dentsply Sirona, NC, USA) using extra-fine quality and default settings. Sintering was performed using a dedicated system (Cerec Speedfire, Dentsply Sirona, NC, USA) that has a patented workflow in terms of temperature and pressure, which allows the whole procedure to be performed in about 15 min. Subsequently, glazing was performed (Celtra universal overglaze, Dentsply Sirona, NC, USA) followed by a second firing procedure, according to the material firing schedules. After visually checking the precision of the restoration on the tooth abutment, all samples were confirmed to be clinically acceptable by two expert operators.

Thereafter, all crowns were pre-treated according to manufacturer instructions, as follows: etching with 5% hydrofluoric acid for 30 s, rinse, ultrasonic alcohol bath for 10 min, dry, primer application (G-Multi Primer, GC Corporation, Tokyo, Japan), dry. On the other hand, teeth were pretreated as follows: cleaning of the surface, selective enamel etching with orthophosphoric acid 35%, rinse, dry, and application of the dedicated functional primer system (G-Cem One Adhesive Enhancing Primer, GC Corporation, Japan). Indeed, even if universal systems can be applied in self-adhesive mode, a dedicated primer was used in the present study. This was made to optimize not only the adhesive properties of the cement but also reduce friction during cementation to improve sitting, as previously shown in other studies [27,28].

The universal dual-curing cement (G-Cem One) was then dispensed, maintaining the tip inside the material to avoid bubble formation, until the crown was filled with the cement. A standardized pressure with a 0.5 kg weight was used during cementation. Samples were randomly allocated (www.randomizer.org (accessed on 8 May 2025)) in two groups (n° = 24 each), according to the light-curing protocol:

- G1: After removing gross excesses with a fine micro-brush (Microbrush^®^ Applicators, Young Innovations Europe, Heidelberg, Germany), 1 min of setting time, light-curing 20 s per buccal, oral, and occlusal sides (total 60 s).
- G2: tack-curing 5 s per side (total 10 s) according to literature and up to reaching a rubbery state [20]. Then, excesses were removed with a new titanium scaler in order to simulate the clinical situation, 1 min of setting time, light-curing 20 s per side (total 60 s).

Light-curing was always performed with a light-emission diode lamp (VALO, Ultradent, South Jordan, UT, USA) at 1400 mW/cm^2^, keeping the light in close contact with the buccal, incisal, and oral surfaces of the tooth. After full polymerization, margins were progressively finished and polished in both groups as follows: red and yellow diamond burs, rubber points, and nylon brush to achieve a glossy surface. Samples were then stored in distilled water at 37 °C in a dark environment. Table 1 reports a summary of used materials alongside their manufacturer and composition.

To investigate marginal adaptation and DC before and after artificial aging, 12 samples per group were investigated at baseline after 24 h from the sample preparation process. The remaining 12 samples per group were treated with thermocycling, according to protocols already presented in the literature [29,30,31]. The protocol consisted of 10,000 cycles in distilled water between 5 °C and 55 °C, with a dwelling time of 1 min each in a dedicated thermocycler (Thermocycler THE-1100, SD Mechatronik, Feldkirchen-Westerham, Germany). To perform Raman Spectroscopy, after micro-CT analysis, the samples were sectioned along the vertical axis in the tooth midline by a 0.33 mm-thick diamond saw. The exposed surface was then finished and polished with ascending grit papers (320 grit, 600 grit, 800 grit, and 1200 grit), and samples were stored in distilled water at 37 °C. The estimation of total sample size for the study setup with three test groups based on α = 0.05 significance level was performed by means of a power analysis with statistical power analysis program G*Power v3.1 (Open Source, HHU) in respect of an estimated effect size of 0.3 and an observed power of 0.85.

### 2.2. Micro-Computed Tomography (Micro-CT) Analysis

Specimens were scanned using micro-CT (SkyScan 1172, Bruker, MA, USA) to evaluate marginal continuity, using the following parameters: voltage = 100 kV; current = 100 μA; aluminum and copper (Al + Cu) filters; pixel size = 10 μm; averaging = 5; rotation step = 0.6°; 180° rotation. To obtain processable files, raw data were reconstructed using NRecon software, Version: 1.7.4.6 (Bruker, MA, USA) with standardized parameters: beam hardening correction = 15%; smoothing = 2; ring artifact reduction = 9. Obtained datasets were saved in Digital Imaging and Communications in Medicine (DICOM) format and imported into a dedicated software (Mimics Medical 24.0, Materialise, Leuven, Belgium) for analysis.

The same procedure was applied after the thermal cycling simulation, maintaining the same parameters to ensure consistency among data. In order to improve the precision of measurements between baseline and after-aging images, which is crucial in linear analysis, all scans were aligned using dedicated software (DataViewer™, Version: 1.4.4, Bruker, MA, USA) using the 3D registration function.

A total of eight slices disposed of in a uniform grill, four in ZY and four in ZX axes, were selected for each sample to obtain sixteen marginal points of analysis, according to the literature [22,32,33,34]. Using the “linear distance” function, all sixteen points were analyzed to calculate external and internal gaps and absolute marginal discrepancy, according to Holmes et al. [35]:

- External gap (EG): perpendicular distance between the external point of the restoration margin and the tooth;
- Internal gap (IG): perpendicular distance between the tooth surface and the crown surface measured in the internal area (0.5 mm from the external point of the tooth margin);
- Absolute discrepancy (AD): distance between the external point of the tooth margin and the external point of the crown margin.

**Figure 1:**
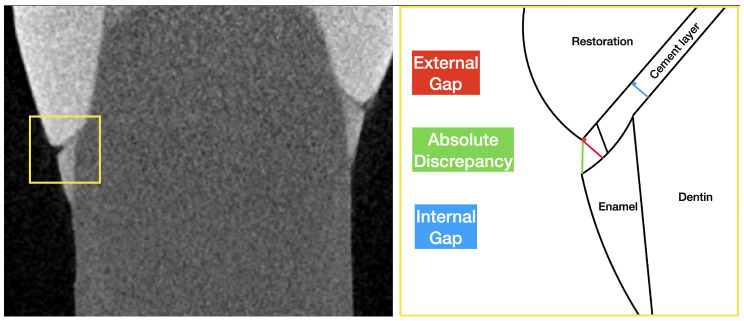
Representative workflow for the linear marginal adaptation analysis. Data are collected in μm for the external gap (red line), internal gap (blue line), and absolute discrepancy (green line). The process is repeated for the 16 points of each sample.

All data were collected as linear distances (μm) and submitted for statistical analysis. A representative workflow is reported in Figure 1 for better understanding.

### 2.3. Raman Spectroscopy Evaluation

A modular Raman spectrometer (B&W Tek, Plainsboro, NJ, USA) equipped with a monochromatic excitation laser (wavelength: 785 nm) and a BTC675N spectrometer (range: 65 cm^−1^–3350 cm^−1^, resolution: 6 cm^−1^), coupled with a CCD sensor was employed to analyze the specimens. The instrument was coupled with the portable BAC151^®^ compact Raman microscope, which enables the analysis of specific areas of the sample, employing several lenses capable of focusing the signal on the surface of the sample. The measurements were carried out using the following parameters: laser power of 100 mW, integration time of 20 s, and 24 repetitions for each area. These parameters were set to maximize the signal/noise ratio and to not induce alteration of the samples [29]. The surface of the sample was scanned with an 80× microscopic objective (spot diameter of about 20 μm) in order to characterize the DC along the cement layer at the cervical, middle, and occlusal areas of the specimens for a total of 5 points for each sample. Uncured specimens of the dual-luting cement were measured and taken as references. The spectra were processed by means of a script written in Python 3.10.12. In particular, the baseline was removed through the asymmetric least squares smoothing and the Savitzky–Golay filter was applied with a window length of 15 cm^−1^. Eventually, a standard normal variate transformation (SVN) was applied: the mean value of the spectra is subtracted from each signal intensity, which is then divided by its standard deviation [36]. Finally, the spectra were narrowed to the region of interest containing the peaks relevant to the DC calculation, i.e., in the range between 1200 cm^−1^ and 1800 cm^−1^.

In order to extract the DC values, it is necessary to monitor the changes of peaks corresponding to specific bonds that undergo a modification during the polymerization process and to compare these bands to the ones that remain unchanged and that can be used as an internal reference. Therefore, in order to calculate the degree of conversion, specific peaks were selected: the peak at 1640 cm^−1^, corresponding to the aliphatic C=C bond, and the peak at 1455 cm^−1^, which is assigned to the C-H bond vibration and that can be used as an internal reference since it remains chemically unchanged throughout the polymerization process [29,37]. The calculation of the degree of conversion is performed based on the following equation (Equation (1))

(1) $$DC\%=\left(1-\frac{R_{\mathrm{cured}}}{R_{\mathrm{uncured}}}\right)\times 100$$

where DC% is the degree of conversion expressed as a percentage, $R_{\mathrm{cured}}$ and $R_{\mathrm{uncured}}$ are the ratio of peak amplitude at 1640 cm^−1^ and the internal reference peak at 1455 cm^−1^ in cured and uncured material, respectively [29,37]. Since the DC was evaluated by comparing the amplitude of the above-mentioned peaks in the spectra, the spectra were further processed with a script developed in Python in order to perform a deconvolution using a Lorentzian function. In particular, once the number and position of the peaks of interest have been defined, the spectra undergo a non-linear fitting based on an incremental least-square optimization algorithm, until the error between the sum of all fitted base peaks and the original spectrum reaches a minimum value. Eventually, the fitted spectra are the results of the sum of the fitted peaks and the parameters of each peak (i.e., center, amplitude, and area).

### 2.4. Statistical Analysis

Marginal continuity data from micro-CT, collected in µm, were imported into Stata 14 software (StataCorp LLC, College Station, TX, USA) for statistical analysis. Since values were normally distributed (Kolmogorov–Smirnov test), two-way ANOVA test was performed to examine the effects of “curing protocol” and “thermal aging”. Post-hoc pairwise comparisons were performed using the Tukey post-hoc test. For all the tests, statistical significance was set at *p* < 0.05. Data obtained by Raman Spectroscopy, on the other hand, were not normally distributed (Kolmogorov–Smirnov test), thus a Kruskal–Wallis test was performed, with significance set at *p* < 0.05.

## 3. Results

### 3.1. Marginal Adaptation by Micro-CT

**Figure 2:**
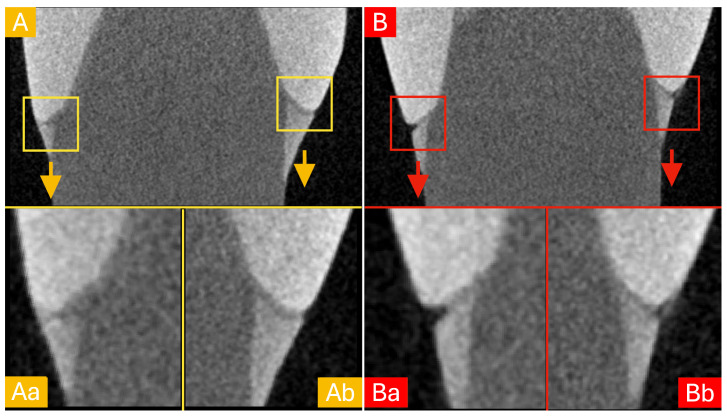
Representative images of a random sample from G1 (**A**) and G2 (**B**). To better assess marginal adaptation, 400% zoom highlights were created in the marginal area. Notice the perfect continuity and absence of defects among the tooth-restoration interface for the G1 sample (**Aa**,**Ab**). On the other side, the sample from G2 presented a cement deficit with a subsequent interfacial gap (**Ba**) and a visible discontinuity in the cement layer (**Bb**).

Representative micro-CT images of the two groups are reported in Figure 2 for a random sample of G1 (Figure 2A) and G2 (Figure 2B). Margins are highlighted in the sub-figures to better appreciate the marginal adaptation. It is worth noticing that samples in group G2 present more discontinuities and some interruptions in the cement layer.

Obtained results of the external gap, internal gap, and absolute discrepancy, expressed as the mean and standard deviation for groups G1 and G2, before and after aging, are listed in Table 2. The two-way ANOVA test showed that the curing protocol significantly influenced the external gap (*p* < 0.01) and absolute discrepancy (*p* < 0.01). Tukey’s post-hoc test revealed that the tack-curing protocol (G2) performed significantly worse (higher discontinuity in the interface). Alternatively, thermal aging had no significant effects on the tested parameters.

### 3.2. Degree of Conversion by Raman Spectroscopy

**Figure 3:**
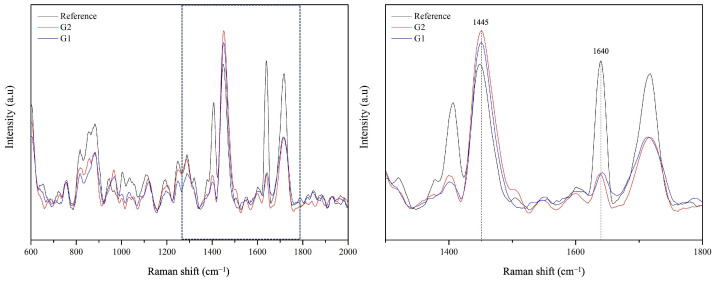
Representative Raman spectra acquired on the uncured cement and after polymerization with the two different curing protocols (G1 and G2). On the left, it is possible to observe the spectra in the range 600 cm^−1^–2000 cm^−1^, while on the right, is a magnified section in the range of 1300 cm^−1^–1800 cm^−1^, with an indication of the peaks of interest.

Representative Raman spectra acquired on the cement before and after polymerization with the two different curing protocols (G1 and G2) are reported in Figure 3. On the right side of the figure, a magnified section of the spectra is shown. The plots evidence the decrease in intensity of the vibrational mode at 1640 cm^−1^ that occurs after polymerization, while the amplitude of the peak at 1445 cm^−1^ is not affected by the light-curing.

**Figure 4:**
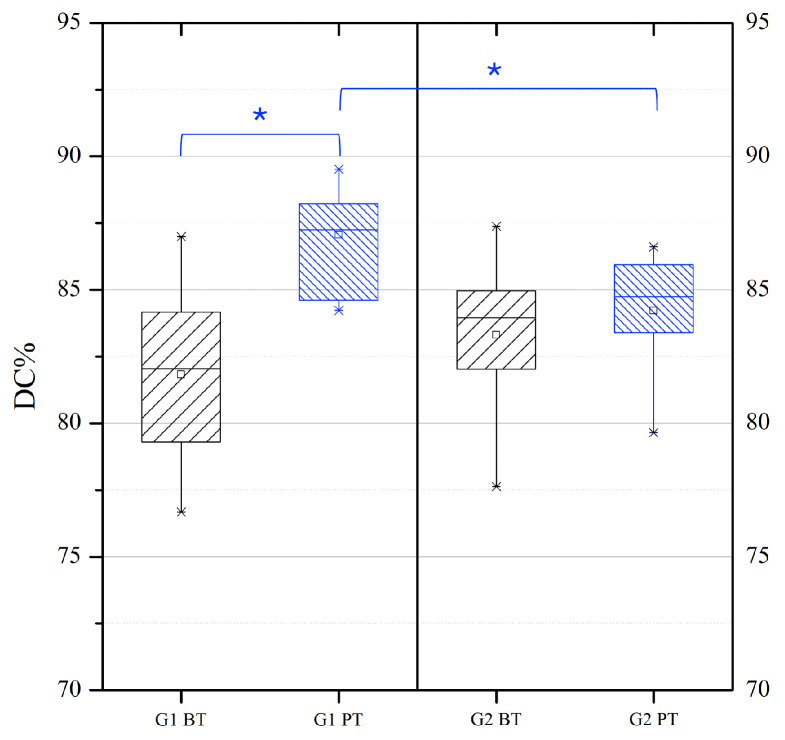
Degree of conversion of luting cement in G1 (black) and G2 (blue) before (BT) and after (PT) artificial thermal aging. The box plots represent the dispersion of the data of the degree of conversion, expressed as a percentage: the boxes include 50% of the DC% values (from 25% to 75%) and are divided by the median line, the square represents the mean value, the bars connect the maximum and the minimum DC% values. The line and asterisks (*) indicate statistically significant differences between groups. The threshold for significance is set at *p* < 0.05.

The box plots in Figure 4 represent the dispersion of the data of the degree of conversion, expressed as a percentage: the boxes include 50% of the DC% values (from 25% to 75%) and are divided by the median line, and the bars connect the maximum and the minimum DC% values. Table 3 reports the obtained results as minimum, maximum, median, mean, standard deviation (std), 25th and 75th percentile values for the degree of conversion expressed as a percentage. It is possible to state that no significant statistical difference between the degree of conversion reached with the two different curing protocols at baseline was detected. On the other hand, the effect of artificial thermal aging led to a statistically significant (*p* < 0.05) increase in the degree of conversion for the specimens in group G1. Nevertheless, the degree of conversion of specimens in group G2 was not significantly affected by the thermal aging treatment. In addition, results showed that there was a significant difference in the degree of conversion data (*p* < 0.05) between the G1 and G2 groups after thermal aging.

## 4. Discussion

Marginal adaptation and degree of conversion of the resin-based restorative materials are key topics in the long-term prognosis of glass–ceramic crowns. The research presented in this paper was, therefore, focused on clarifying the effect of two different curing protocols on the marginal continuity and the degree of conversion of a universal dual-curing cement under full-ceramic crown restoration before and after artificial aging. To assess margin continuity in the present study, X-ray micro-computed tomography (micro-CT) was selected. Micro-CT has been proven to be very effective for this purpose, thanks to high resolution and the possibility to match scans before and after aging [38]. This was confirmed by a recent review by Contrepois et al., which defined micro-CT as the only method that allows both a precise identification of critical gaps and definition of margin conditions [4].

The selected micro-CT workflow has been proven to efficiently assess the interfacial integrity, even if limited to the analyzed slices [39]. Although the definition of “clinically acceptable” has changed several times in the last decades, it is generally accepted that a mean marginal gap ranging in the interval of 50 μm–120 μm is sustainable for restorations cemented with resin-based materials [40,41,42,43]. In the present study, according to the obtained results, all specimens showed a marginal adaptation in this range.

Concerning micro-CT results, the first null hypothesis could be partially accepted since the marginal integrity is significantly affected by the curing protocol but not by the artificial aging process. The conventional curing protocol group (G1) showed a significantly better external gap and absolute discrepancy compared to the tack-curing group (G2). The underperformance of tack-curing could be related to the uneven mechanical removal of cement excesses with the scaler, above all when an incomplete setting of the cement has been obtained. According to Freire et al., 1 s tack curing does not affect marginal adaptation. However, longer tack-curing times, in the range of 3 s–5 s, which are necessary to let the excess obtain a jelly phase and make it removable through a scaler, can potentially result in nonuniform areas in terms of DC [9]. This can lead to heterogenous viscosity of the cement and consequently variable efficiency of the scaler on the marginal section. Even though it does not compromise physical proprieties, this might cause damage to the interface continuity, especially if under- or over-contours are present [44,45].

With regard to aging effects on micro-CT adaptation, no significant differences were reported. These results agree with other studies, in which the thermocycling showed no statistical influence on marginal adaptation [46,47]. A higher number of thermal cycles in combination with mechanical loading should be assessed to provide further information about the artificial aging effect on the interfacial sealing ability of a universal resin-based luting cement. Vibrational spectroscopy techniques find great application in the study of a variety of materials, including metals, ceramics, polymers, composites, and several tooth tissues. Since it may shed light on both the chemical features of the materials under research and the chemical bonds and reactions that take place during the polymerization process, vibrational spectroscopy is particularly well suited for the investigation of materials used in restorative dentistry as dual luting cements. As a matter of fact, RS is frequently used for dental materials evaluation since it can be utilized for gathering data with quick and non-invasive methods, providing spectra with distinctive bands that are assigned to molecule bonds and that can be used to identify and characterize the material. RS has been used to examine the DC of resin-based composites, adhesive systems, and luting cements [48,49,50,51]. Many indirect methods, such as microhardness, have been employed to indirectly assess the degree of conversion of restorative materials; however, methodologies based on RS have the advantage of providing a direct assessment of the DC [48,49,50,51]. Indeed, DC can be found by observing changes in the shape or intensity of specific peaks connected to chemical bonds that are modified by the polymerization process. Specifically, the size or intensity of the peak at 1640 cm^−1^, associated with the C=C vibrational mode, can be compared to a reference band before and after the polymerization process to determine the DC value.

Upon Raman spectroscopy analysis, the second null hypothesis could be partially accepted since the curing protocol did not affect the DC, but the artificial aging test provided a significant increase. In any case, it was observed that the material reached a satisfactory DC, with values greater than 70% for all samples in both groups G1 and G2, with no statistically significant differences based on the polymerization process. Indeed, the DC should be greater than 60% to be considered clinically acceptable, namely, to reach the chemical and mechanical properties that can assure the longevity of indirect restorations [52]. It is worth noticing that this result was achieved even if the dual luting cement was exposed to light curing through the ceramic crown, proving the applicability of such material for indirect restoration, as reported in [53]. However, it is worth noticing that the DC of the material changes after artificial aging for group G1, which could be attributed to various factors. Indeed, even though the maximum values of DC are usually reached during the first 30 min after light activation, dual luting cements could present a subsequent gradual increase in DC [54]. It must be considered that dimethacrylate-based dental resin composites could undergo initial immobilization of the monomers during the early stages of polymerization, which may lead to a subsequent slower polymerization process even after light exposure, regardless of thermocycling [55,56,57,58]. The early vitrification indeed depends on several factors, such as the composition and the filler content of the cement, which is around 70 wt% for the G-Cem One universal dual-curing cement [51]. Another factor to be considered is that the initial fast polymerization involved in the tack-curing method (G2) could immobilize the polymer network with the first formed bonds in a more extended way with respect to the traditional curing method (G1) leading to more pronounced changes in G1 samples when the material is then exposed to thermocycling. Indeed, the DC only provides information regarding the number of bonds that have changed, and not on the polymer network that is formed. Thermocycling may have enhanced the mobility and collision frequency of the residual reactive species, such as free radical and unreacted monomers, resulting in a further polymerization process if the structure of the network formed in the G1 group was more regular since there was no initial fast polymerization [53,54,55,56,57,58,59].

## 5. Conclusions

This study combined micro-computed tomography and Raman spectroscopy to evaluate two polymerization protocols for a novel dual-curing luting cement. Micro-CT revealed that tack-curing (G2) exhibited heterogeneity in the restoration and material detachment, likely due to incomplete initial polymerization. In contrast, both groups achieved a degree of conversion above 70%, as confirmed by Raman spectroscopy. Artificial aging did not cause visible structural changes, but Raman analysis showed increased conversion degree in G1, proving its sensitivity to molecular-level changes. These results emphasize the importance of selecting appropriate curing protocols and demonstrate the value of combining macro- and micro-level assessment methods.

Although tack curing offers clinical convenience, it was associated with marginal defects that could compromise restoration longevity. These interfacial gaps are particularly dangerous due to the inherent vulnerability of adhesive restorations at their margins. Therefore, within the limitations of this in vitro study, conventional polymerization and finishing techniques are advisable to minimize such risks. Study limitations include the limited number of thermal aging cycles (10,000); more extended or aggressive thermal cycling protocols could provide further insights into long-term performance. Additionally, the use of a single, experienced operator, while minimizing variability, may limit the generalizability to less experienced clinicians. Furthermore, the study focused on a single, relatively new universal dual-curing cement with specific protocols; future research should investigate other materials and varying tack-curing timings to check similar outcomes.

Future studies will explore long-term performance under mechanical loading and the impact of different types of ceramic crowns on the marginal adaptation and degree of conversion of the cement. In addition, future work will investigate post-polymerization development of the degree of conversion, with a specific emphasis on the study of polymerization and aging kinetics.

## Figures and Tables

**Table 1:** Summary of employed materials, alongside their manufacturer and composition.

Material	Manufacturer	Composition
Universal dual-curing cement (G-Cem One)	GC, Tokyo, Japan	Paste A: Fluoroaluminosilicate glass, urethane dimethacrylate (UDMA), dimethacrylate, initiator, stabilizer, pigment, silicon dioxide, 10-methacryloyloxydecyl dihydrogenphosphate (10-MDP).
		Paste B: SiO_2_, trimethoxysilane, UDMA, 2-hydroxy-1,3-dimethacryloxypropane, 10-MDP, 6-tert-butyl-2,4-xylenol, 2,6-di-tert-butyl-p-cresol, ethylenediaminetetraacetate (EDTA) disodium salt dehydrate, vanadyl acetylacetonate, 2,4,6-Trimethylbenzoyldiphenylphosphine oxide (TPO), ascorbic acid, camphoroquinone, Mg.
Dedicated primer (G-Cem One Primer)	GC, Tokyo, Japan	Ethanol, MDP 4-methacryloxyethyl trimellitic anhydride (4-META), 2-hydroxy-1,3-dimethoxypropane, vanadyl acetylacetonate, 2,6-di-tert-butyl-p-cresol.
Reinforced lithium silicate (Celtra Duo)	Dentsply Sirona, NC, USA	Lithium silicate with 10% ZrO_2_.

**Table 2:** Gap and discrepancy measurements before and after aging.

Group	External Gap (µm)		Internal Gap (µm)		Absolute Discrepancy (µm)	
	Baseline	After Aging	Baseline	After Aging	Baseline	After Aging
G1	87.79 ± 40.86	94.22 ± 41.57	61.07 ± 26.80	65.88 ± 29.50	92.94 ± 35.41	100.76 ± 46.16
G2	108.73 ± 38.37	115.74 ± 37.05	68.37 ± 21.55	70.27 ± 21.43	117.92 ± 45.93	128.21 ± 42.73

**Table 3:** Minimum, maximum, median, mean, standard deviation (std), 25th and 75th percentile values for the degree of conversion expressed as percentages according to the different curing protocols (group 1, G1 and group 2, G2) and thermal aging (BT: before thermocycling, PT: post thermocycling).

Statistic	G1 BT	G1 PT	G2 BT	G2 PT
Minimum	76.68	84.23	77.63	79.65
Maximum	87.00	89.52	87.38	86.61
Median	82.05	87.46	83.96	84.91
Mean	81.83	87.06	83.31	84.22
Std	2.82	1.86	2.77	2.23
25th percentile	79.58	86.02	82.03	83.55
75th percentile	84.09	88.29	84.96	85.84

## Data Availability

The original contributions presented in this study are included in the article. Further inquiries can be directed to the corresponding authors.

## Abbreviations

The following abbreviations are used in this manuscript:

DC	Degree of Conversion
GC	Glass–Ceramic
RS	Raman Spectroscopy
CEJ	Cement–Enamel Junction
CAD	Computer-Assisted Design
